# Research Progress on the Application of Topological Phase Transition Materials in the Field of Memristor and Neuromorphic Computing

**DOI:** 10.3390/s23218838

**Published:** 2023-10-30

**Authors:** Runqing Zhang, Rui Su, Chenglin Shen, Ruizi Xiao, Weiming Cheng, Xiangshui Miao

**Affiliations:** School of Integrated Circuits, Huazhong University of Science and Technology, Wuhan 430074, China; m202272804@hust.edu.cn (R.Z.); ruisu@hust.edu.cn (R.S.); u202114554@hust.edu.cn (C.S.); m202172796@hust.edu.cn (R.X.); miaoxs@hust.edu.cn (X.M.)

**Keywords:** topotactic phase transformation, memristor, signal conversion, non-volatile memory, synapses

## Abstract

Topological phase transition materials have strong coupling between their charge, spin orbitals, and lattice structure, which makes them have good electrical and magnetic properties, leading to promising applications in the fields of memristive devices. The smaller Gibbs free energy difference between the topological phases, the stable oxygen vacancy ordered structure, and the reversible topological phase transition promote the memristive effect, which is more conducive to its application in information storage, information processing, information calculation, and other related fields. In particular, extracting the current resistance or conductance of the two-terminal memristor to convert to the weight of the synapse in the neural network can simulate the behavior of biological synapses in their structure and function. In addition, in order to improve the performance of memristors and better apply them to neuromorphic computing, methods such as ion doping, electrode selection, interface modulation, and preparation process control have been demonstrated in memristors based on topological phase transition materials. At present, it is considered an effective method to obtain a unique resistive switching behavior by improving the process of preparing functional layers, regulating the crystal phase of topological phase transition materials, and constructing interface barrier-dependent devices. In this review, we systematically expound the resistance switching mechanism, resistance switching performance regulation, and neuromorphic computing of topological phase transition memristors, and provide some suggestions for the challenges faced by the development of the next generation of non-volatile memory and brain-like neuromorphic devices based on topological phase transition materials.

## 1. Introduction

With the rapid development of the information age, the internet, artificial intelligence (AI), big data, and other fields have developed significantly, bringing about an explosive growth of various types of data. However, the increasingly large amount of data on the hardware arithmetic requirements is also increasingly high; the existing means struggle to meet the needs of such data-intensive applications. Traditional computers use the Von Neumann architecture, which separates storage and computation; the CPU must first read data from the storage unit when executing commands, and then save the computed results back to the storage unit. The repeated transmission of data between the CPU and memory will cause high latency and huge power consumption, and the development of the CPU’s data processing speed is becoming faster, but the memory’s data access speed lags behind. This memory bottleneck makes it difficult for high-performance CPU to play its due role, which limits the improvement of the overall computing efficiency and leads to the emergence of the Von Neumann bottleneck and “Memory Wall”. Unlike computers based on the traditional Von Neumann architecture, the neural system of the human brain utilizes neuronal and synaptic activity to simultaneously process and store information, and there is no clear boundary between the two. The neuromorphic architecture of the human brain, on the other hand, is characterized by parallelism, self-adaptation, and self-learning [1], which is conducive to handling the increasingly large data processing demands. This enables a wide range of applications in fields such as automatic driving, image recognition, and artificial intelligence. Among the various devices that simulate neurons and synapses, memristors are highly favored because they can simulate synaptic characteristics well and have great application prospects.

Memristors were first proposed by Prof. Chua of the University of California, Berkeley, in 1971, as the fourth type of primary source circuit component to characterize the relationship between electric charge and magnetic flux from the perspective of completeness of circuit theory. They were named “memristors” [2]. A memristor is a nonlinear resistor whose resistance can vary with the current and voltage flowing through it, and that reversibly switches between at least two stable resistance states in response to an applied electrical signal. However, in the three decades since the theory was proposed, research on memristors has focused more on the basic theory of memristors, without much progress on device implementation. In 2008, Strukov et al. from HP Labs prepared a Pt/TiO_2_/Pt stacked device and showed a hysteresis loop with resistive switching properties using voltage scanning [3], demonstrating the true practical realization of a memristor and the possibility of determining whether it is a memristor or not by the presence of a hysteresis loop.

One of the important applications of memristors is as a new type of non-volatile memory, i.e., resistive random-access memory (RRAM), due to its unique memristive characteristics. Compared with other traditional memories, the resistive change memory has a storage density comparable to Flash, an operating speed comparable to DRAM, and also combined with low power consumption and non-volatility. In addition, synapses in a neural network can change synaptic weights through the transmission of neurotransmitters between pre- and postsynaptic neurons, whereas a memristor regulates the conductance value of a device by applying bias voltages to the top and bottom electrodes. The conductance values of the device and the synaptic weights correspond to each other and are transformed into each other, so that the synaptic function can be perfectly simulated by the memristor. Utilizing this new type of memory device can realize data storage while performing computation, which means integrating storage and computation and fundamentally eliminating the supposed memory bottleneck. Based on this, more research groups have started to engage in the research of memristors in neuromorphic computing, and some breakthroughs have been achieved [4,5,6].

The common structure of the memory device is a “sandwich” structure composed of metal/insulating layer/metal, which is a vertically stacked structure, that is, a functional layer with a resistive switching effect is sandwiched between two layers of metal or other electrode materials. In addition, there is a horizontal structure of the memristor, which is common in the application of two-dimensional materials. The device electrodes are horizontally arranged on the surface of the functional layer. Because of the migration of defects or the structural phase transition in 2D materials [7,8], the device will produce resistive switching. In particular, in horizontal memristors, the functional layer is generally directly exposed, so it is easy to perform in situ measurements. However, for the topological phase transition materials with three-dimensional perovskite structures, the devices are basically dominated by vertically stacked structures.

The functional layer of the memristor is usually an insulator with a large resistance. Various types of electrode materials and functional layer materials jointly affect the resistive switching mechanism and resistive switching performance of the device. Functional layer materials for memristors are generally categorized into two main types, that is, organic materials and inorganic materials. Organic functional layer materials have giher costs and a simpler preparation process; inorganic materials mainly include chalcogenides, binary oxides, and multiple oxides. Currently, binary metal oxides are the most widely used, such as HfO_x_ [9,10,11], TiO_x_ [12,13,14], and AlO_x_ [15,16,17], whose resistive switching behaviors are mainly dependent on the formation and fracture of the oxygen vacancy conductive filament (CF) in the oxides, and that have the characteristics of low-power consumption and compatibility with the CMOS process, thus having promising potential in industrial production. It is found that many perovskite-type oxide materials also exhibit non-volatile, low-power consumption, fast switching speed, and excellent memristor characteristics, which can be well applied to the field of memristors. In particular, perovskite-structured topological transition materials have attracted extensive attention from researchers because of their unique topological phase transition properties with a clear and controllable resistive switching mechanism. This paper mainly discusses the research progress of topological phase transition materials, including the resistive switching mechanism, the resistive switching performance regulation, and the application of topological phase transition memristors in neural networks. Finally, the development trend and potential application prospects of topological phase transition memristors are also pointed out.

## 2. Resistive Switching Behaviors in Topological Phase Transition Memristors

(1) Topological phase transition materials

As it is well known, in perovskite materials, variations in the oxygen stoichiometry can have a significant impact on the structure and properties of the materials. Among these, non-stoichiometry in oxygen can lead to the formation of oxygen vacancies within the structure, providing rapid migration pathways for oxygen ions. This leads to the emergence of interesting properties, such as significant changes in the material’s conductivity and oxygen ion mobility at high temperatures. These properties demonstrate potential applications in fields such as energy and information storage.

Perovskite-structured transition metal oxides (TMOs) have attracted extensive attention due to their fast and anisotropic oxygen ion transport properties. Among these, the most typical structure is the perovskite structure with topological phase transition properties, known as the brownmillerite (BM) structure. The term “topological phase transition material” refers to a material in which the lattice symmetry undergoes distortion when a portion of its constituent elements is extracted. However, if the overall framework of the material remains stable despite this distortion, the material is referred to as a topological phase transition material.

Common topological phase transition metal oxide (TMO) materials include iron-based materials (e.g., SrFeO_x_(SFO) [18]), cobalt-based materials (e.g., LaCoO_x_ [19,20], SrCoO_x_(SCO) [21,22], and La_1-x_Sr_x_CoO_x_ [23,24]), and manganese-based materials (e.g., La_x_Sr_1-x_MnO_x_ [25,26], and SrMnO_x_ [27,28]). Typically, controllable arrangements of oxygen vacancies in the materials can be achieved through methods such as annealing in high-temperature vacuum (or oxygen) atmospheres [29], ion liquid gating [30], and electron beam irradiation [31]. These approaches lead to reversible transitions between oxygen-rich and oxygen-deficient states. Notably, strontium ferrite (SrFeO_x_) and strontium cobaltite (SrCoO_x_) exhibit higher oxygen migration rates, and their Gibbs free energy differences for the topological phase transition from brownmillerite (BM) to perovskite (PV) phase are smaller than other TMO materials [32], which makes low-temperature phase transition possible. Furthermore, SrCoO_x_ theoretically possesses the lowest overpotential compared with other perovskite materials [33], which is favorable for oxygen evolution reactions [34]. Therefore, it is widely believed that SrFeO_x_ and SrCoO_x_ hold significant practical application prospects.

**Figure 1:**
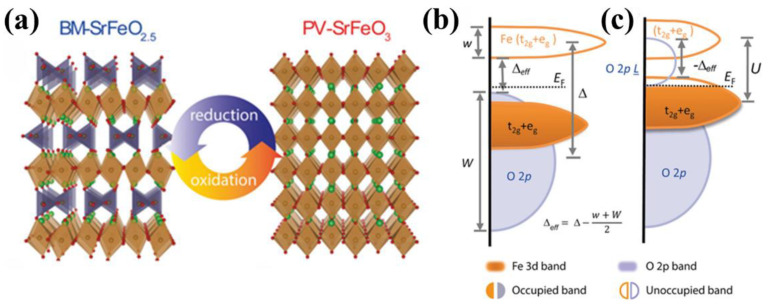
(**a**) Lattice structures of SrFeO_2.5_ brownmillerite (BM) and SrFeO_3_ perovskite (PV), (**b**) band structure of BM-SFO, and (**c**) band structure of PV-SFO [39]. Copyright 2019, Wiley-VCH Verlag GmbH & Co.

In the SrFeO_x_ system, a reversible transition occurs between the SrFeO_2_._5_(BM-SFO) Brownmillerite phase and the SrFeO_3_(PV-SFO) Perovskite phase by gaining or losing a fraction of oxygen. At room temperature, the crystal structure and band structure of SFO are illustrated in Figure 1. The crystal structure of BM-SFO consists of alternating FeO_4_ tetrahedra and FeO_6_ octahedra, while the PV-SFO is composed solely of stacked FeO_6_ octahedra. Apart from the crystal structure, BM-SFO and PV-SFO also exhibit significant differences in their band structures. In BM-SFO, the valence band is comprised of O 2p orbitals, and the conduction band is formed by Fe 3d orbitals. The charge transfer energy is positive, resulting in a 2 eV band gap, classifying the BM phase as an insulating state [35,36]. Upon introducing oxygen ions into BM-SFO, Fe^3+^ is converted to Fe^4+^, and the material turns into the PV phase. An electron from the O 2p orbital is transferred to the Fe 3d orbital, leaving an unfilled O 2p state in the middle of the band gap. This forms a hole state, leading to a negative charge transfer energy and, thus, PV-SFO becomes a conductive phase. Notably, the Fe^4+^ in PV-SFO does not exhibit a simple d^4^ configuration; rather, it takes a d^5^L configuration (where L represents ligand holes), which is quite similar to the d^5^ configuration of Fe^3+^ in BM-SFO, which might contribute to the transition between the two topological phases [37,38]. The combination of small Gibbs free energy differences, negative charge transfer energy, and lower oxygen vacancy formation energy makes SFO a potential candidate for multifunctional device applications.

In practical applications, for better control over the performance of resistive switching devices based on SFO, it is essential to achieve epitaxial growth of the SFO functional layer, which helps in avoiding the introduction of uncontrollable defects due to experimental conditions. As a result, the preparation of SFO generally employs pulsed laser deposition (PLD) techniques. Additionally, other perovskite materials with topological phase transition characteristics have also captured the attention of numerous researchers. For instance, La_0.7_Sr_0.3_MnO_3_, which possesses a perovskite structure, can undergo a reversible topological phase transition under specific conditions. However, its insulating BM phase exhibits a strong oxygen affinity, and the transition to the conductive PV phase does not necessarily require oxygen-rich conditions. This stands in contrast with cobalt-based and iron-based TMOs, highlighting its potential applications in solid oxide fuel cells or oxygen sensors [40]. In the following parts, we will discuss the research progress in resistive switching devices based on these multi-component perovskite-type topological phase transition materials.

(2) Study of Resistance Switching Effects in Topological Phase Transition Memristors

In general, the conduction mechanisms of memristors can be broadly categorized into the electrochemical metallization mechanism (ECM), the valence change mechanism (VCM), the thermochemical mechanism (TCM), and the pure electron effects. Regarding perovskite structure topological phase transition materials, as described in the previous explanation of their crystal structure and band characteristics, applying a positive or negative bias voltage at room temperature to topological phase transition materials (taking SrFeO_x_ and SrCoO_x_ as examples) causes the accumulation (or repulsion) of oxygen ions within the material, resulting in the formation of an oxygen-rich conductive region (or oxygen-deficient insulating region) and consequently the formation of non-metallic CFs in the functional layer. This process induces a change in the valence state of the metal elements, constituting the VCM.

In particular, when a Schottky barrier exists between the electrode and the functional layer, the aggregation or repulsion behavior of oxygen ions (or oxygen vacancies) leads to changes in the interface barrier height, thereby influencing the resistive switching effects of the device. Another phenomenon is observed when there are BM or PV layers within the functional layer. Upon applying a voltage, the whole interface of the BM or PV layers shifts, resulting in a change in the device’s resistance. Devices exhibiting resistive switching effects based on such conductive mechanisms are referred to as Interface-type Memristors. Therefore, topological phase transition memristors can be primarily classified into filament-type and interface-type devices. In the following sections, we will focus on the resistive switching effects and related research progress of these two types of devices.

In fact, researchers started studying cobalt-based and iron-based TMO materials relatively early. As far back as 2011, research groups investigated SCO materials [41], although at that time the focus was predominantly on the magnetic properties of SCO, and less on the electrical property changes originating from the material’s topological phase transition. In 2013, Jeen et al. [42] utilized PLD to deposit BM-SCO and SrCoO_3−δ_ thin films on (001)-oriented SrTiO_3_ (STO) and (001)-oriented (LaAlO_3_)_0.3_–(SrAl_0.5_Ta_0.5_O_3_)_0.7_ (LSAT) substrates. They found that these two phases could exhibit a rapid and reversible transition at significantly reduced temperatures (200–300 °C) within a minute. This low-temperature and fast reversible transition was attributed to the low Gibbs free energy difference between the two phases. They further introduced a new ion material, SCO, that could potentially be applied in the field of information storage.

**Figure 2:**
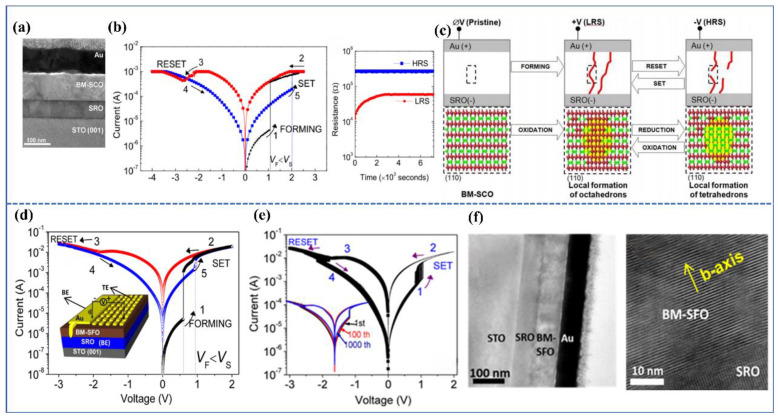
(**a**) Cross-sectional TEM image of the Au/BM-SCO/SrRuO_3_/SrTiO_3_(001) device, showing clear boundaries between layers. (**b**) I–V curve of the Au/BM-SCO/SrRuO_3_/SrTiO_3_(001) device within a voltage scanning range of −4V to 3V, exhibiting resistive switching characteristics of the CF type and retention behavior. The device shows stable high-resistance behavior but instability in the LRS during the initial stage. (**c**) Schematic diagram of the filament model as the resistive switching mechanism (top) and the topologically distinct phase transition of SCO induced by external bias-induced reversible redox reactions (lower) [43]. Copyright 2014, AIP Publishing. (**d**) Schematic diagram of the Au/BM-SFO/SrRuO_3_/SrTiO_3_ (001) device structure and I–V cycling characteristics. (**e**) I–V curves for 100 consecutive cycles of set and reset processes. (**f**) Cross-sectional TEM image of the Au/SrFeO_x_/SrRuO_3_/SrTiO_3_(001) device and cross-sectional TEM image near the interface between BM-SFO and SrRuO_3_ thin films [47]. Copyright 2016 American Chemical Society.

By 2014, Tambunan et al. [43], through PLD, prepared SrRuO_3_ bottom electrodes and BM-SCO functional layers on strontium titanate (SrTiO_3_) substrates. They fabricated devices with square-shaped gold (Au) top electrodes (Au/BM-SCO/SrRuO_3_/SrTiO_3_(001)) using lithography and electron beam evaporation techniques. Cross-section transmission electron microscope (TEM) observations of the device revealed relatively smooth interfaces between the functional layers and electrodes, as shown in Figure 2a. Subsequently, they tested the I–V characteristics of the devices within the voltage scanning range of 0 V→2.5 V→0 V→−2.5 V→0 V. The I–V characteristic curve, displayed in Figure 2b, demonstrates the device’s bipolar resistive switching behavior, where the resistance switching is related to the magnitude and polarity of the scanning voltage. An initial forming process was required during the initial scan. When the voltage reached *V*_set_, the device transitioned from a high-resistance state (HRS) to a low-resistance state (LRS). When the voltage was reversed to *V*_reset_ during the voltage scan, the device reverted from LRS to HRS. Notably, they found that the lower *V*_forming_ than *V*_set_ reflected an unconventional CF formation process. Additionally, the device exhibited certain fatigue resistance and retention characteristics. Moreover, by altering the size of the top electrode, they observed variations in high and low resistance and changes in high and low resistance values with temperature. They discovered that the resistive switching unit in the LRS was composed of a mixture of BM-SCO and PV-SCO phases. The transition from BM-SCO to Mix-SCO exhibited bipolar resistive switching behavior in a localized manner, which was attributed to the phase transition effect of SCO.

Researchers have established a corresponding model for the internal CF mechanism in SCO, as depicted in Figure 2c. In the initial state, no CF exists within the functional layer, placing the device in an insulating state. Upon applying a forming voltage, the device undergoes a soft breakdown operation, resulting in the emergence of certain CFs within its interior, connecting the top and bottom electrodes. Consequently, the resistance of the device suddenly decreases. When the voltage scans towards the negative polarity, the oxygen ions forming the CFs within the functional layer are repelled and migrate towards the SrRuO_3_ bottom electrode, leading to the rupture of the CFs. The device’s resistance then abruptly increases, leaving behind numerous CoO_4_ tetrahedral clusters at the previously formed CF locations, which causes the device to return to HRS. At this point, the device’s resistance is lower than its initial resistance (as the CFs in the functional layer have not completely disappeared). If the voltage is again scanned towards the positive polarity, compared with the initial case where oxygen ions only needed to fill a layer of CoO_4_ tetrahedra, a larger positive voltage is required to attract more oxygen ions to fill the increased number of CoO_4_ tetrahedra, forming vertical CFs, which results in higher *V*_Set_ than *V*_forming_. During subsequent voltage scanning processes, the device begins to alternate between LRS and HRS, then achieving resistive switching.

This report marked the first time that a topological phase transition memristor based on the CF mechanism was introduced. Its structure is a simple “sandwich” configuration, and the fabrication process is not complex. In previous reports, PLD technology has been extensively used for the surface deposition of complex compound materials. This technique displays many advantages, including accurate film composition, high deposition rate, good film quality, and high uniformity. Therefore, many researchers have adopted PLD technology to deposit topological phase transition TMO materials [44,45,46].

Acharya and Nallagatla [47] conducted similar research on SFO. They fabricated Au/SrFeO_x_/SrRuO_3_/SrTiO_3_(100) memristive devices, as shown in the inset of Figure 2d. The I–V characteristics of these devices are similar to those of the devices shown in Figure 2a, exhibiting bipolar resistive switching with smaller *V*_F_ than *V*_S_. This unique resistive switching behavior can be explained using a model similar to Figure 2c. The difference is that the stability of these devices is improved. As shown in Figure 2e, after the initial forming process, the devices exhibit excellent cyclic stability, enabling high- to low-resistance cycles for up to 1000 cycles. This is in contrast with the earlier SCO devices, which could only cycle about 30 times. Acharya attributes this difference to the fact that the prepared SCO films cannot guarantee atomic-level flatness on their surfaces. Consequently, many areas near the top electrode with locally minimal thickness could preferentially form CFs, leading to non-uniform CF paths. In this work, epitaxial SFO thin films can grow with atomic-level flat surfaces, as shown in Figure 2f, resulting in improved uniformity of the devices.

Through the aforementioned discussions, we can understand that for topological phase transition materials such as SCO or SFO, the memristors function by controlling the migration of oxygen ions within the functional layer through the application of electrical stimuli at the device electrodes, thereby achieving resistance switching. A characteristic feature is the abrupt change in resistance observed in the I–V hysteresis loop when the voltage reaches a transition threshold. This corresponds to the formation or rupture of CFs within the functional layer. However, from previous research, direct evidence of the presence of CFs has been lacking, with only some indirect indications suggesting their potential existence. For instance, these indications include examining the relationship between resistivity and temperature to infer the composition of the thin film or altering the size of the electrodes to discern effects (wherein electrode size has a greater impact on high-resistance values compared with low-resistance values) indicative of filament-based devices. Consequently, the quest for tangible proof confirming the existence of CFs has become a focal point for an increasing number of researchers.

**Figure 3:**
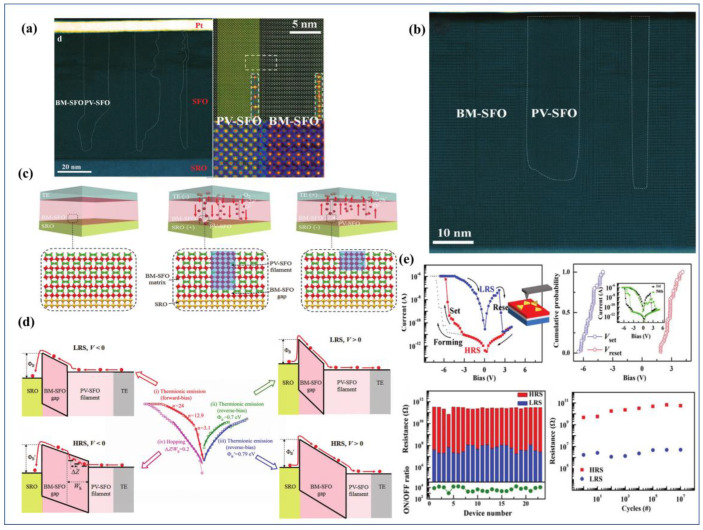
(**a**) TEM image of the large-sized Pt/BM-SFO/SRO device in LRS, distinguishing and directly observing the presence of PV-phase CF through Fast Fourier Transform (FFT), where the CFs do not directly connect the top and bottom electrodes. (**b**) TEM image in HRS, depicting a fracture in CF segment b, yet it remains connected to the top electrode. (**c**) Schematic illustration of the conduction mechanism in the Pt/BM-SFO/SRO device. (**d**) Conduction mechanism fitting using the I–V curve and description of the Schottky barrier at the interface between the CF and the bottom electrode, indicating that the Schottky barrier height varies with the BM-SFO gap width. (**e**) I–V characteristic curves of the Au/SFO/SRO nano-device, cumulative probability plot of *V*_set_ and *V*_reset_ over 50 I–V cycles, measured R_HRS_ and R_LRS_ for 23 non-memristive units, corresponding switching ratios, and the fatigue resistance characteristic curve of the device [48]. Copyright 2019 Wiley-VCH Verlag GmbH & Co.

It was not until 2019 that Tian et al. [48] devised a simple Pt/BM-SFO/SRO device and directly observed the presence of CFs. Testing its I–V characteristics, the device initially exists in an insulating state and requires a forming voltage to undergo a soft breakdown. Notably, this forming voltage is of negative polarity, and |*V*_Reset_| < |*V*_Set_| < |*V*_forming_|. Upon applying a positive voltage to the electrode, a distinct change in resistance can be readily observed, indicating the rupture of CFs at this point. To offer direct evidence for the presence of CFs, the researchers employed aberration-corrected scanning transmission electron microscopy (AC-STEM) to examine SFO films in different states (LRS and HRS). As depicted in Figure 3a,b, it can be observed that the formed CFs extend from the Pt electrode towards the bottom electrode, which aligns with the results of negative-polarity forming. When subjected to −|*V*_forming_|, oxygen from the surrounding air is drawn into the functional layer, initially filling the oxygen vacancies in the BM-SFO near the top electrode, before gradually diffusing towards the bottom to form the PV-phase CFs. It is worth noting that in LRS, CFs do not establish a complete connection between the top and bottom electrodes as previously conceived, rather, a 6.6 nm gap of BM-SFO exists between the CFs and the bottom electrode. This gap could be attributed to BM-SFO’s incomplete insulation, making it unable to withstand sufficiently high voltage to induce its transition to the PV phase. Subsequent mechanistic fitting by the authors revealed the presence of a Schottky barrier (Φ_B_) between the bottom electrode and CFs, with its height correlating with the width of the BM-SFO gap. When the width is narrow, the barrier height is low, allowing electrons to easily overcome the barrier, thus making the BM-SFO gap less insulating. Consequently, the device remains conductive overall. With an increase in gap width, Φ_B_ also increases, leading to electrons traversing the gap and encountering traps, thereby emphasizing the dominance of hopping conduction.

Subsequently, Tian et al. engineered Au/SrFeO_x_/SrRuO_3_ nano-devices based on nanoscale PV CFs (approximately 10 nm), depositing nanoscale triangular metal electrodes on the thin film, as depicted in Figure 2e. Testing revealed that these devices exhibited excellent uniformity, with the on/off ratio reaching 10^4^, significantly surpassing that of large-sized devices. Furthermore, these devices demonstrated high durability over 10^7^ cycles. These advantages are likely attributed to the lower number of CFs within the nanoscale device units, which largely mitigates the issues arising from the incomplete rupture of multiple CFs, leading to low resistance and poor stability. Through investigation of nanoscale memristive devices, it was indicated that SFO material holds immense potential for high-density information storage devices.

In summary, we can deduce from the above discussions that the use of transmission electron microscopy can aid in distinguishing the superlattice structure of the BM phase from the typical perovskite structure of the PV phase, which can enhance the clarity of the conduction mechanism of TMO topological phase transition memristors based on the perovskite structure. This distinction also highlights the differentiation of this material from traditional binary oxides. At present, some binary and ternary TMOs memristors have achieved a good resistive switching performance. For example, in the study of asymmetric TaO_x_ resistive switching devices, Lee et al. [49] found that the asymmetric device has a lower SET voltage (~1 V), higher switching speed (~10 ns), and ultra-high endurance (>10^12^ cycles), which provides a favorable reference for the high-density integration of memristors. In addition, in the study based on a ZnO_x_ memristor, Yang et al. [50] found that the device had a low SET voltage (~1 V), a large on/off ratio (~10^7^), a very high switching speed (~5 ns), and an ultra-long retention time (>10^7^ s), showing an ultra-fast and highly scalable memory element. For the study of ternary TMOs, Nili et al. [51] reported a memristive device based on amorphous SrTiO_3_. This device has non-volatile bipolar resistive switching characteristics, a very high switching ratio (10^3^–10^4^), and good endurance (>10^6^ cycles), showing the prospect of amorphous perovskite memristors in memristive systems.

However, traditional binary metal oxides such as AlO_x_ and TiO_x_ often suffer from non-uniform distribution of intrinsic defects in thin films due to limitations in fabrication processes [52], leading to random formation and rupture of oxygen vacancy filaments in devices, impacting their uniformity [53]. Additionally, except for some memristors based on CFs made of metals like Ag and Cu, most memristors involving binary oxide CFs like HfO_x_ and TaO_x_ cannot be directly observed [54], impeding further investigation into their resistance-switching mechanisms. Therefore, the perovskite-structured TMO topological phase transition material is deemed to be an ideal candidate for memristor materials.

**Figure 4:**
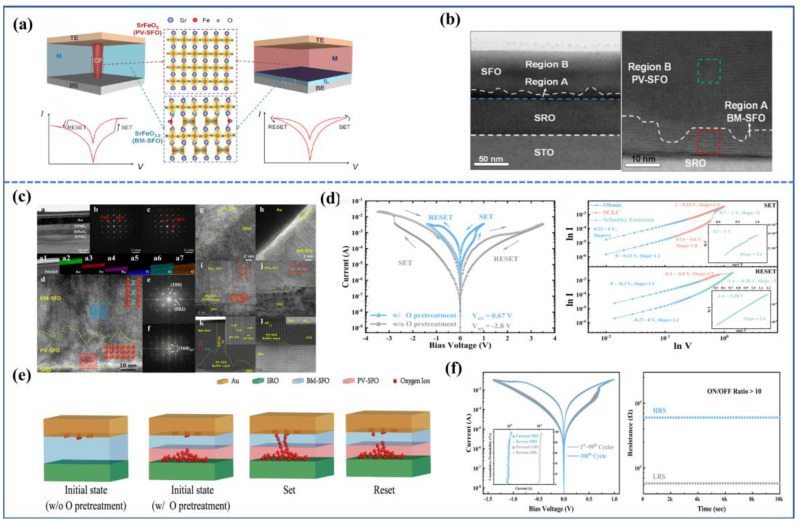
(**a**) Schematic diagrams and I–V characteristic distinctions between filament-type and interface-type memristors. (**b**) Cross-sectional TEM image of the Au/PV-SFO/BM-SFO/SrRuO_3_/SrTiO_3_ device [63]. Copyright 2021, Elsevier Ltd. (**c**) Cross-sectional TEM image of the Au/BM-SFO/PV-SFO/SrRuO_3_/SrTiO_3_ device, clearly depicting the presence of an approximately 20nm PV-SFO buffer layer. (**d**) I–V characteristic curve and corresponding fitted conduction mechanism curve of the Au/BM-SFO/PV-SFO/SrRuO_3_/SrTiO_3_ device. (**e**) Resistive switching illustration of the Au/BM-SFO/PV-SFO/SrRuO_3_/SrTiO_3_ device. (**f**) Accumulated I–V characteristic curves during 100 cycles and retention characteristics of the device [64]. Copyright 2022, Elsevier Ltd. and Techna Group S.r.l.

In addition to the aforementioned memristive devices based on CF mechanisms, there exists another type of device that does not require the formation of a CF connecting the top and bottom electrodes. Instead, it relies on the overall migration of the conductive interface, causing changes in the interface potential barrier and subsequently altering the device resistance. This type of device is known as an interface-type device. It is worth noting that in such devices, the resistance (or conductivity) changes continuously during the resistive switching process, as opposed to the abrupt resistance change caused by the formation or rupture of a CF in filament-type memristors, as shown in Figure 4a. Moreover, interface-type devices typically do not require an initial electroforming process, and thus lead to lower power consumption, faster operation speed, and greater practical applicability. However, compared with filament-type memristors, research on interface-type devices is relatively limited, with most studies focused on binary oxides such as TaO_x_ [55,56] and TiO_x_ [57,58]. For example, Ryu et al. [59] reported a self-rectifying Ti/TiO_2_/HfO_2_/Si interface-type memristor, which exhibits good endurance characteristics (>1000 cycles) and retention characteristics (>10^4^ s), as well as more resistance states (~49) to simulate synaptic weights, and because the self-rectifying behavior enhances the interface switching performance, it is better to apply the simulation of artificial neural networks. In addition, Kunwar et al. [60] found that the Au/Nb: STO interface-type device showed good simulated resistive switching characteristics, a large on/off ratio (10^5^), and good retention (10^4^ s) and endurance (10^2^ cycles) characteristics. The device can successfully simulate various biological synaptic functions such as excitatory postsynaptic current (EPSC), paired-pulse facilitation (PPF), long-term potentiation/depression (LTP/LTD), and spike timing dependent plasticity (STDP), which proves that the interface-type memristor device can be used to develop highly reliable synaptic devices for neuromorphic computing. Therefore, research on topological phase transition materials with good crystallinity, high oxygen migration rates, and oxygen migration anisotropy is of significant interest.

In fact, in the preparation of the interface-type topological phase change memristor, it is generally necessary to form a dielectric layer rich in oxygen ions or oxygen vacancies, and then indirectly affect the oxygen concentration at the interface of the functional layer. This method of introducing oxygen vacancies in the film preparation process is a very important method for non-destructively regulating the oxygen content. Some research groups have also achieved good results using this method. For example, when studying HfO_2_-based memristors, Peng et al. [61] introduced a BiFeO_3_ oxygen vacancy layer in the double HfO_2_ layer to improve the poor conductivity modulation linearity of the original device. This behavior of the oxygen vacancy layer introduced during the device fabrication process makes the formation of CFs easier (meaning lower forming voltage), and promotes the gradual oxidation and reduction of CFs during the set and reset processes, thereby improving the linearity of LTP/LTD, which is beneficial to the device’s synaptic simulation and application in neural networks. For the research of controlling the oxygen vacancy content in the functional layer during the fabrication process, Pyo et al. [62] regulated the oxygen content of amorphous Pr_0.7_Ca_0.3_MnO_3_ (PCMO) films grown on TiN/SiO_2_/Si (TiN-Si) substrates by controlling the oxygen pressure during deposition. Moreover, they found that the device fabricated under low oxygen pressure contained more oxygen vacancies, which was more conducive to the formation of CFs. The resistive switching process does not require the forming process and exhibits a multi-resistance state, which is more conducive to the application in neural networks.

In 2021, Rao et al. [63] fabricated an Au/PV-SFO/BM-SFO/SrRuO_3_/SrTiO_3_ device, where the functional layer is not a single BM-SFO or PV-SFO layer, but rather a PV/BM-SFO heterostructure. As depicted in Figure 4b, the cross-sectional view of the device reveals that the dominant phase is PV-SFO within the functional layer, while the BM-SFO layer is located near the interface with the bottom electrode and has a thickness of only about 10 nm. The formation of the oxygen-deficient BM-SFO layer can be attributed to the presence of oxygen vacancies on the surface of the SrRuO_3_ bottom electrode. During the deposition of the SFO functional layer, oxygen ions in the vicinity of the bottom electrode migrate downward to fill the oxygen vacancies at the interface, resulting in the appearance of the BM phase. Through conductive atomic force microscopy (C-AFM) scanning, it was observed that applying a positive voltage significantly enhanced the conductivity within the scanned area, and the conductive regions were uniformly distributed, indicating interface-type resistive switching behavior. During device operation, applying a positive *V*_set_ voltage caused oxygen ions within the SrRuO_3_ to migrate into the functional layer, leading to a reduction in the thickness of the BM-SFO layer. This allowed electrons to traverse through the BM-SFO layer and thus increased conductivity. Additionally, due to the non-uniform nature of the BM-SFO layer, some electrons were able to tunnel through thinner gaps (without Schottky barrier emission conduction), resulting in enhanced conductivity in specific regions of the interface. Applying a reset voltage prompted a substantial migration of oxygen ions from the PV/BM interface towards the SrRuO_3_ bottom electrode, causing an increase in BM-SFO thickness and higher potential barrier height. Consequently, the electrons were difficult to tunnel through further, and favored Schottky emission or hopping conduction. This behavior is reflected in the smooth transition between high and low resistance observed in the I–V characteristic curve, demonstrating a continuous change in resistance (conductance), which is conducive to simulating synaptic functions and subsequent applications in neural networks.

However, not every PV/BM-SFO heterostructure necessarily constitutes an interface-type device. Su et al. [64] fabricated a device with a PV-SFO buffer layer, Au/BM-SFO/PV-SFO/SrRuO_3_/SrTiO_3_, using a unique oxygen pre-treatment process. The device structure is illustrated in Figure 4c, and Fourier transform techniques were employed to distinguish between PV and BM-SFO structures. They discovered the presence of an approximately 20 nm PV-SFO buffer layer near the bottom electrode in the device structure. The dominance of the BM-SFO layer and the orientation of a superlattice stripe pattern, which is favorable for oxygen migration, were confirmed. Notably, through mechanism fitting, it was determined that the conduction mechanism of this device adhered to the Schottky emission conduction of incompletely connected PV-SFO CFs, rather than the interface-type mechanism, as suggested by Rao et al.

We suggest that the divergence in conduction mechanisms arises from varying oxygen ion content in the functional layer. In Rao et al.’s device, PV-SFO is the primary component, and a significant amount of oxygen ions exist in the functional layer. Consequently, when operating voltages are applied to the top electrode, a collective migration of a large number of oxygen ions at the BM/PV interface occurs, leading to a substantial change in BM-SFO layer thickness. Conversely, in Su et al.’s device, the functional layer is predominantly BM-SFO phase. As a result, under *V*_set_, there are not enough oxygen ions for collective migration at the BM/PV interface. Instead, CFs connecting to the top electrode are formed only at the interface. However, the path for CF is shorter, leading to a significantly reduced *V*_set_ compared with the original device. Under *V*_reset_, even though oxygen ions from the PV buffer layer may migrate to SRO, decreasing the PV-SFO thickness, electrons still cannot overcome or tunnel through the Schottky barrier. Consequently, in this scenario, the device operates mainly through the filament conduction mechanism.

In summary, there is currently no definitive consensus on the physical mechanism driving the resistive switching effect in topological phase transition memristors. The aforementioned discussion presents two of the most typical mechanisms. Thus, the investigation of the physical mechanism of topological phase transition memristors remains a focal point for future research.

## 3. Modulation of Topological Phase Transition Memristor Performance

The performance of topological phase transition memristors typically manifests in two aspects. Firstly, for their application in the field of information storage, memristor performance primarily encompasses attributes such as the on/off ratio, operating voltage, uniformity among device units, retention characteristics, switching speed, endurance, and more. Secondly, they find application in simulating neural synapses, specifically mimicking the long-term potentiation (LTP) and long-term depression (LTD) characteristics of biological synapses. These features necessitate continuous enhancement or reduction of the device’s conductivity under polarity-alternating continuous pulses. Different application scenarios require varying performance criteria, motivating researchers to enhance memristor performance.

The structure of topological phase transition memristors typically constitutes a sandwich structure composed of electrodes and a functional layer. The electrodes mainly facilitate the transmission of electrons between the electrode and functional layer, thereby exerting a considerable influence on the overall device performance. Presently, research on electrodes primarily centers around the top electrodes. This is because the functional layer of topological phase transition memristors requires epitaxial growth, which usually occurs on a perovskite structure bottom electrode through epitaxial techniques. Consequently, bottom electrodes like SrRuO_3_ or La_0.7_Sr_0.3_MoO_3_ are commonly employed, leaving limited room for structural modifications. However, recent studies, like Rao et al. [63], controlled oxygen pressure during the deposition of the bottom electrode, leading to the presence of oxygen vacancies at the SrRuO_3_ surface, consequently forming a BM-SFO interface layer, thereby influencing device performance. In contrast, the top electrode does not have clear requirements for shape, size, or type, rendering it a viable method for modulating device performance.

**Figure 5:**
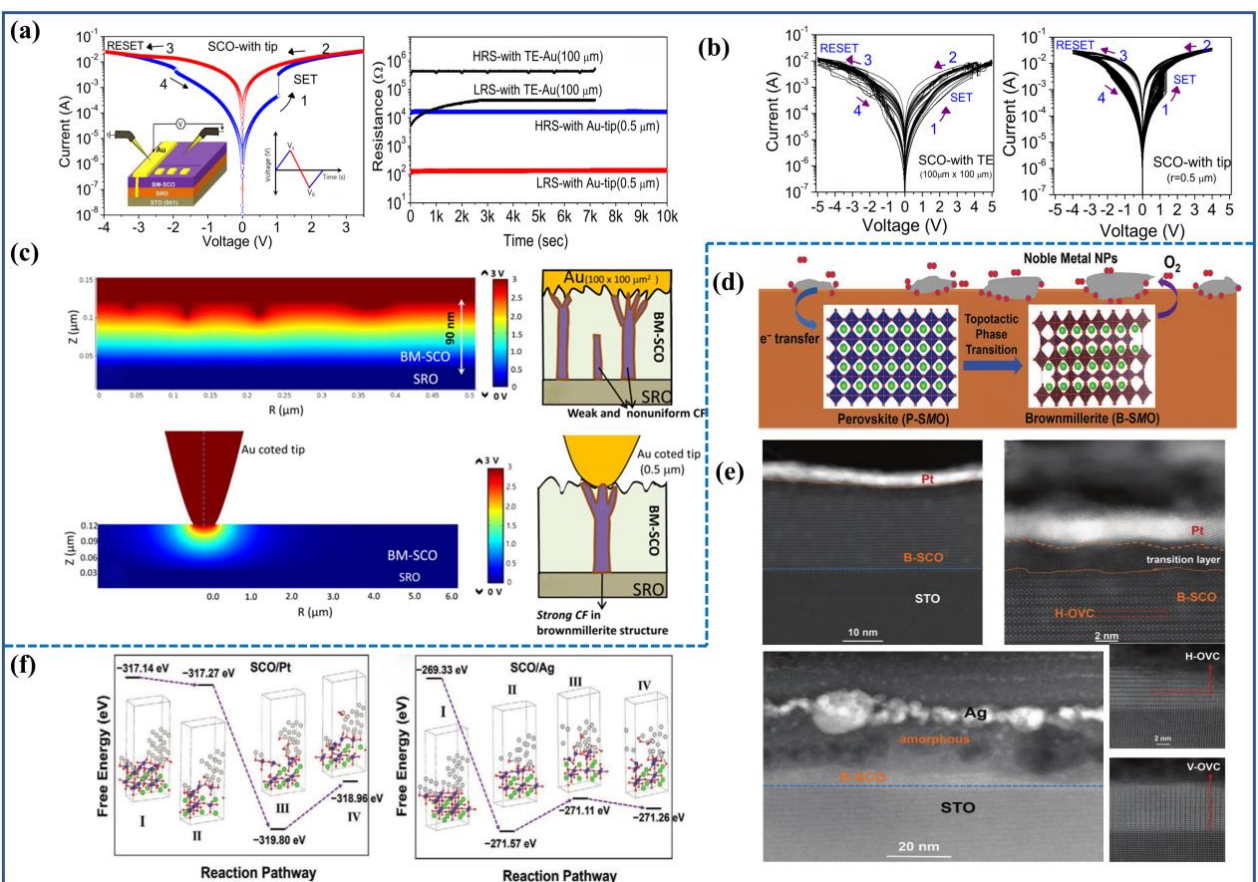
(**a**) Structure, I–V characteristics, and retention properties of Au/BM-SCO/SrRuO_3_/SrTiO_3_ devices with different electrode sizes. (**b**) Cycling curves of Au/BM-SCO/SrRuO_3_/SrTiO_3_ devices with different electrode sizes under 30 voltage scans. (**c**) Finite element simulation of electric field distribution within the functional layer after applying voltage for different electrode sizes and schematic of resistive switching in the device [56]. Copyright 2019, Springer Nature. (**d**) Schematic of noble-metal-assisted deoxygenation process in PV-SCO and PV-SFO. (**e**) Schematic of resistive switching in Au/PV-SFO/BM-SFO/SrRuO_3_/SrTiO_3_ device. (**f**) Dynamic change of free energy during PV-SCO deoxygenation process under different states using DFT calculations [57]. Copyright 2021, Wiley-VCH Verlag GmbH & Co.

One aspect of electrode modulation involves controlling electrode size. For functional layer films with uneven surfaces, electrode size has a substantial impact on their performance. Nallagatla et al. [65] prepared a Au/BM-SCO/SrRuO_3_ device, as shown in Figure 5a, which exhibited clear filament conduction switching behavior. The device employed a square-shaped top electrode with dimensions of 100 μm. As a control group, gold-coated probes were used in place of top electrodes for testing (the contact area between the probe and the film is approximately 0.5 μm). Testing results revealed that LRS of the large electrode device was challenging to maintain. After the SET process completed, the device’s resistance gradually increased over time, and eventually held. Moreover, the switching ratio and stability of the large electrode device were lower than those of the small electrode device, as depicted in Figure 5b. To explain this phenomenon, finite element simulations were conducted to visualize the electric field distribution within the functional layer following applied voltage, as shown in Figure 5c. The simulation results revealed that due to the uneven film surface, applying voltage to the large electrode resulted in localized regions with concentrated electric fields, leading to a gathering of numerous oxygen ions. This facilitated the formation of multiple randomly distributed CF formation sites, promoting the development of numerous weak conduction filaments. Oxygen ions in these filaments could disperse at any time, leading to the resistance decay shown in Figure 5a. In the reset process, inconsistent filament ruptures would cause unstable *V*_set_ in subsequent cycles, consequently affecting overall device stability. Conversely, for the small electrode device, finite element simulations revealed that the electric field at the electrode-film interface was more concentrated. This stronger attraction of oxygen ions promoted strong CF formation and reduced the number of CFs per cell, resulting in a higher switching ratio and stability.

Furthermore, even for atomically flat interfaces, electrode size still affects device performance. Indeed, in large electrode devices, the phenomenon of CF-preferred formation sites is absent, resulting in improved device stability. However, the high resistance of the device is significantly influenced by the electrode size, while the low resistance is less affected, so the overall on/off ratio is relatively small. Nano-sized devices, as prepared by Tian et al. [48], exhibit enhanced switching ratios and improved cycling stability, as shown in Figure 3e. In the future, it may be possible to engineer CF formation sites through controlling the electrode contact area or shape on flat interfaces, allowing for more precise modulation of the resistive switching performance.

Influencing another aspect of the top electrode is the effect of electrode material; Wang et al. investigated the impact of different metal top electrodes on SCO and SFO topological phase transition materials [66]. They deposited two noble metals (Pt, Ag) onto SrCoO_3-δ_ perovskite oxide films annealed under high oxygen pressure using electron beam evaporation. Combined with first-principle density functional theory calculations (DFT), they found interactions involving surface charge transfer and cooperative bonding between Pt, Ag, and SCO. These interactions weakened the Co-O (Fe-O) bonds and lowered the energy barrier for oxygen migration, making the migration of oxygen ions within the thin film more active and facilitating the topological phase transition. The role of noble metal electrodes is depicted in Figure 5d, where noble metals transfer electrons to the topological transition material, forming Co (Fe)-O-noble metal (Nm) bonds that prevent the direct rupture of Co (Fe)-O bonds. This promotes the diffusion of oxygen ions to the surface, ultimately converts to O_2_, indicating that noble metal layers can act as oxygen pumps to continuously extract oxygen from the oxygen-rich film to the atmosphere. Additionally, the film transforms from PV phase to BM phase without bias voltage at low temperatures, and completes the topological phase transition.

Interestingly, the two electrodes also exhibit significantly different extraction strengths of oxygen from the film, as shown in Figure 5e. For the Pt electrode, except for the formation of a 4 nm conversion layer due to a rapid oxygen migration at the surface, the rest of the region consists of horizontal stripe-like BM-SCO. In contrast, the Ag electrode shows a different behavior, with most areas becoming amorphous and non-crystalline due to the uncontrolled release of oxygen. Only a small amount of BM-SCO can be observed near the substrate, and both horizontal (H-OVC) and vertical (V-OVC) stripe-like structures exist simultaneously. Combined with DFT calculations, the free energy change trends for noble metal-induced oxygen migration in different states can be observed. States I→II→III represent the migration of oxygen within the thin film and the electrode, while state III→IV represents the deoxygenation process of forming O_2_. Regardless of whether it is Pt or Ag electrodes, the overall free energy of oxygen from the initial state to the final deoxygenation state decreases, which explains why the topological phase transition can occur without additional energy input at room temperature. However, for SrFeO_3-δ_ (0 < δ < 0.5), Fe-O bonds are stronger than Co-O bonds and have a higher deoxygenation barrier. Although the first and second steps of oxygen atom migration are energetically favorable, the overall deoxygenation process involves an increase in entropy and requires heat absorption. Therefore, to complete the entire process, the temperature needs to be raised to 150 °C, which is much lower than the temperature required for phase transition without noble metal electrodes (700 °C). Thus, by deliberately selecting different electrode materials, the concentration of oxygen ions within the film can be influenced, thereby modulating the resistive switching performance of the memristor.

**Figure 6:**
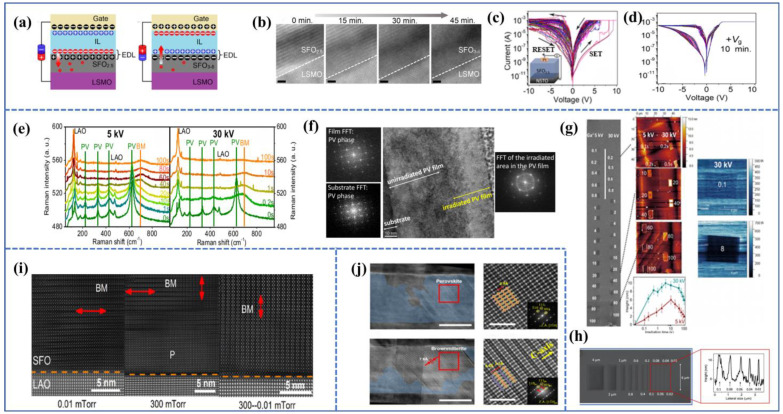
(**a**) Schematic cross-sectional images of LSMO/BM-SFO samples under negative and gated voltages. (**b**) In situ TEM images of BM-SFO thin film under negative gate bias (−2 V) with time variation. (**c**) I–V characteristic curve of Pt/BM-SFO/NSTO device without IL gating. (**d**) I–V characteristic curve of device after applying positive IL gating [67]. Copyright 2019, American Chemical Society. (**e**) Raman spectroscopy spectra under ion radiation at 5 kV and 30 kV at room temperature. (**f**) Cross-sectional TEM image of PV-SFO after high-dose Ga^+^ focused ion irradiation. (**g**) SEM, AFM, and C-AFM images of PV-SFO irradiated with Ga^+^ ions at different doses (in seconds) at 5 kV and 30 kV energy levels. (**h**) SEM and AFM morphology images of PV-SFO film after 30 kV Ga-FIB irradiation [68]. Copyright 2020 AIP Publishing. (**i**) Cross-sectional TEM images of SFO thin films deposited on LaAlO_3_ under different oxygen pressures and subsequent annealing conditions [70]. Copyright 2019, AIP Publishing. (**j**) High-resolution transmission electron microscope (HR-TEM) image of the SrFeO_3_(111) device (light blue area corresponds to perovskite region, and dark striped area corresponds to brownmillerite region) along with the corresponding Fast Fourier Transform (FFT) [74]. Copyright 2020, AIP Publishing.

Another common method of controlling the oxygen ions within the film after the fabrication of memristor devices is ion liquid gating (IL gating). Saleem et al. conducted a detailed study on the influence of oxygen-rich ionic liquid on BM-SFO films [67], as illustrated in Figure 6a. They employed N,N-diethyl-N-(2-methoxyethyl)-N-ethyl ammonium bis-(trifluoromethyl sulfonyl)-imide as IL, which covered the entire film, and Al electrodes were used. After gate operation, the sample was washed with acetone and ethanol to remove IL. When a negative voltage (−*V*_g_) is applied to the Al electrode, an electric field is formed in the IL, driving oxygen ions in the IL to migrate into BM-SFO and it increases the concentration of oxygen ions within the film. Conversely, when a positive voltage (*V*_g_) is applied to the electrode, oxygen ions are extracted from the film and memristor device, and it restores the initial state. The impact of IL gating on the film structure can be observed through in situ TEM observations, as shown in Figure 6b. Initially, the BM-SFO film exhibits horizontal stripe-like structures. After applying negative IL gating and continuously acting for 45 min, the superlattice stripes structures gradually disappear from the SrFeO_x_/LSMO interface, likely due to the rapid oxygen vacancy diffusion along the horizontal superlattice channels within the IL during in situ observation. To investigate the effect of IL gating on the film’s electrical properties, Pt/BM-SFO/NSTO devices were prepared using (0.7 wt.%) Nb-doped (001) oriented SrTiO_3_ (NSTO) substrates. The initial resistance of the device is about 3 GΩ, and a large operating voltage is required to lower the device’s resistance. After multiple cycles, the device’s stability is found to be poor. Continuous IL gating is then applied to the device. With prolonged exposure, the initial resistance, *V*_set_, and switching behavior of the device decreases, while the *V*_reset_ increases before stabilizing. The IL gating mechanism is as follows: the initial concentration of oxygen ions within the device is low, necessitating a large *V*_set_ for the formation of CFs through oxygen ion aggregation. The participation of oxygen ions in the migration process is minimal, leading to instability in the I–V curve of the device. After applying negative IL gating, oxygen ions enter the film, and the film gradually transforms towards the PV phase, which results in a decrease in initial resistance. Additionally, the size of CFs in LRS is large and CFs are also liable to form at a low *V*_set_. Conversely, when the device is brought back to HRS during the negative voltage cycle, large CFs are less likely to be disrupted, leading to a higher *V*_reset_.

It is evident that IL gating is an effective way to adjust the resistance switching (RS) parameters. This method’s advantage lies in its ability to control the concentration of oxygen ions after the completion of device fabrication. As the IL gating process is relatively compatible with device and battery integration processes, it holds significant potential for practical applications.

In fact, for SrFeO_3-δ_ thin films, there is a more convenient and rapid method to induce its topological phase transition. Ferreiro-Vila et al. investigated the effects of low-dose Ga^+^-focused ion irradiation on the topological phase transition of SrFeO_3-δ_ thin films [68]. By comparing the effects of Ga^+^ irradiation at different energies, it was found that at 30 kV, the optimal resolution was achieved due to the minimization of lens aberrations. Furthermore, using Ga^+^ irradiation at 5 kV and 30 kV on SrFeO_3-δ_ thin films, as shown in Figure 6g, it was observed through SEM, AFM, and C-AFM tests that after Ga^+^ focused ion irradiation, the lattice of irradiated region expanded due to the formation of BM-SFO (confirmed by Raman spectroscopy), leading to a decrease in conductivity. The expansion was most pronounced at 30 kV energy, and as the dose (expressed in seconds) increased, ion irradiation also caused milling effects. This explains the reduction in film thickness and the occurrence of film amorphization at high irradiation doses (typically above 40 s), as shown in Figure 6f. Therefore, low-dose Ga^+^ irradiation at 30 kV was employed in this study. Considering the lattice expansion characteristic of SFO transitioning from the PV to BM phase, another advantage of FIB technology is its application for high-resolution nano-patterning. As depicted in Figure 6h, by controlling the width of Ga^+^ irradiation, distinct elongated regions were revealed in the SEM image (width ranging from 4000 nm to 20 nm), which shows the instructive potential of ion irradiation techniques for the design and utilization of future nano-devices.

From the previous introduction of topological phase transition materials, it is known that the perovskite structure (taking BM-SFO as an example) consists of alternating stacking of FeO_4_ tetrahedra and FeO_6_ octahedra, resulting in an insulating phase. In TEM measurements, alternating bright and dark stripes can be observed, with the dark stripes representing the so-called “superlattice stripes”. The direction of these superlattice stripes can be considered as the fastest direction of oxygen migration [69]. Therefore, theoretically, adjusting the direction of superlattice stripes could effectively regulate the device’s performance.

Wang et al. successfully prepared BM-SFO thin films on LaAlO_3_ (LAO) substrates with superlattice stripes oriented perpendicular to the in-plane direction by controlling the oxygen pressure during film deposition and annealing processes [70]. Figure 6i shows TEM images of SFO films deposited under different oxygen pressure conditions. Under low oxygen pressure deposition conditions, a horizontal OVC structure was preferentially formed at the interface, and the grown film contained excess oxygen that could not escape to the vacuum, resulting in less noticeable superlattice stripes. For films grown under high oxygen pressure (300 mTorr), a perovskite PV-SFO structure was preferentially formed at the interface. However, as no annealing was performed, areas far from the SFO/LAO interface would exhibit the coexistence of horizontal and vertical OVCs. For films deposited under high oxygen pressure and annealed under low oxygen pressure, oxygen was released preferentially along the vertically aligned OVCs in a way that oxygen in PV-SFO was released, and the vertical OVCs gradually replaced the horizontal ones. Therefore, after the deposition and annealing processes, vertically aligned OVCs were formed from top to bottom. Subsequent testing of the optical band gap for different films revealed that as the “purity” of BM-SFO increased, the optical band gap increased and the conductivity decreased. Currently, research on vertically oriented pseudo-superlattice stripes in topological phase transition materials is more concentrated in fields of optics [71] and magnetism [72], with relatively few studies in electronics, especially in the field of resistive switching devices. An example is the device developed by Su et al. [64], which featured a Au/BM-SFO/PV-SFO/SRO/STO structure. As shown in Figure 4d, the device with vertically oriented pseudo-superlattice stripes exhibited significantly lower operating voltages than those with horizontally oriented pseudo-superlattice stripes. Additionally, the vertically oriented device displayed excellent cycle stability and retention characteristics (Figure 4f). The significantly reduced operating voltage can be attributed to two factors: the 20nm PV-SFO buffer layer shortening the path for CF formation and the vertical OVC enhancing oxygen ion migration efficiency in the out-of-plane direction. This substantial reduction in operating voltage is beneficial for the development of low-power devices.

Furthermore, apart from controlling oxygen pressure during film deposition, the orientation of superlattice stripes can be manipulated by selecting different crystallographic orientations of the substrate. Acharya et al. fabricated Au/BM-SFO/SrRuO_3_/SrTiO_3_ (111) devices and compared their performance with devices fabricated on (100) oriented crystallographic substrates under the same conditions, investigating the influence of crystallographic orientation on device performance [73]. Generally, (100) oriented substrates tend to promote the growth of BM-SFO/BM-SCO thin films with an in-plane oxygen vacancy channel (H-OVC), while (111) oriented substrates favor the growth of BM-SFO/BM-SCO thin films with vertically oriented oxygen vacancy channel (V-OVC), where the angle between the FeO_4_ tetrahedra and FeO_6_ octahedra layers and the substrate is 55°. For the Au/BM-SFO/SrRuO_3_/SrTiO_3_(111) device performance testing, it was found that the device required a negative forming process, and after soft breakdown, the device was in an intermediate state between high resistance and low resistance. When the voltage was scanned to the positive *V*_set_, the device switched to LRS, exhibiting good retention characteristics, cycle stability, and maintaining good endurance under positive and negative pulse stimulation. Compared with the SrFeO_x_ (100) device, the (111) device also demonstrated better stability between devices. This improved stability in the (111) device is possibly due to the oxygen vacancy channel connecting the top and bottom electrodes, which helps reduce the randomness in CF formation.

On the other hand, Kim et al. utilized in situ TEM to observe structural changes during the resistive switching process of Au/BM-SFO/SrRuO_3_/SrTiO_3_(111) and Au/BM-SFO/SrRuO_3_/SrTiO_3_(001) devices, analyzing the effects of crystallographic orientation on the resistive switching performance [74]. During sample testing, Au served as the ground, and the scan voltage was applied on SrRuO_3_. The most significant difference in performance between the two types of devices is that the (001) device requires a negative *V*_forming_ to initiate a certain level of CF formation, whereas the (111) device, due to its inherent oxygen vacancy channels connecting the top and bottom electrodes, allows for easier oxygen ion migration under the electric field, rendering the soft breakdown process unnecessary. The actual in situ voltage testing confirmed that the I–V curves of both devices matched theoretical expectations. TEM observations revealed that in the (111) device, a large area of PV-SFO was formed in LRS, and most of the PV phase disappeared after the transition to HRS, transforming into the BM phase. In contrast, for the (001) device, after the initial forming process, air’s oxygen entered BM-SFO, filling the oxygen vacancies in the material. This process led to the formation of PV-SFO in certain regions and transformed the device into HRS. As oxygen ion migration across the octahedral layers is challenging, subsequent set and reset processes mainly occurred near the vicinity of the Au electrode. The (111) device exhibited a higher oxygen ion migration rate along the electric field direction compared with the (001) device. Consequently, the (111) device had more oxygen ions participating in migration during the resistive switching process, leading to a larger area of resistive switching. On the other hand, the (001) device had a smaller switching region. These works clearly demonstrate that selecting substrate crystallographic orientation can control the orientation of superlattice stripes, which contributes to the improvement of resistive switching device performance based on BM phase topological phase transition materials.

**Figure 7:**
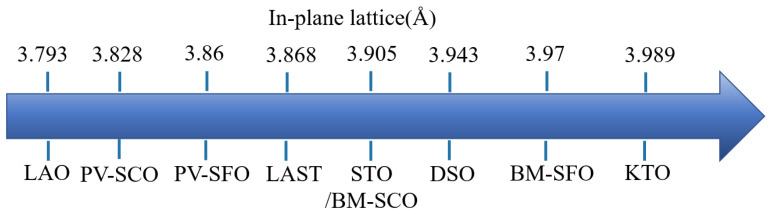
In-plane lattice constants of several common substrates: LAO (LaAlO_3_), LSAT ((LaAlO_3_)_0.3_–(SrAl_0.5_Ta_0.5_O_3_)_0.7_), STO (SrTiO_3_), DSO (DyScO_3_), KTO (KTaO_3_), PV/BM-SFO, and PV/BM-SCO [79,80,81].

In addition to controlling pseudo-superlattice stripe orientation by adjusting the substrate crystallographic orientation, another approach involves inducing in-plane stress by selecting substrates with different lattice constants from the thin films. The in-plane lattice parameters of common substrates and SFO/SCO are shown in Figure 7. Previous studies have shown that the activation energy of oxygen vacancies in conventional perovskite structure materials can be affected by epitaxial strain. Through first-principle calculations, it was found that 4% tensile strain can reduce the formation energy of oxygen vacancies by about 0.4 eV compared with the unstrained materials [75] (note that the epitaxial growth-induced strain should be less than 5%). However, for materials with high oxygen vacancy formation energies (>2 eV) grown under epitaxial strain, the effects of strain on the formation energy are relatively insignificant. Interestingly, some materials like SCO and SFO exhibit particularly low oxygen activation energies (<1 eV) [76,77,78], which magnifies the impact of strain on oxygen vacancy formation energy. Consequently, tuning the performance of topological phase transition resistive memory devices through strain manipulation has become a research hotspot.

**Figure 8:**
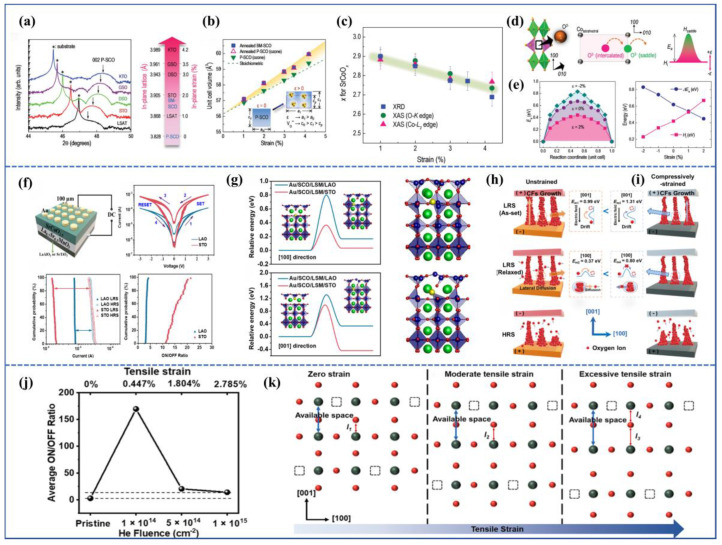
(**a**) XRD patterns of PV-SCO films deposited on different substrates. (**b**) Variation of unit cell volume of different oxygen stoichiometry SCO films with tensile strain. (**c**) Variation of x in SCO with tensile strain. (**d**) Schematic illustration of oxygen ion migration from one vacancy site to another and the relationship between activation energy E_a_, enthalpy of oxygen insertion H_i_, and saddle point H_saddle_. (**e**) Variation of activation energy E_a_ with strain [82]. Copyright 2016, Wiley-VCH Verlag GmbH & Co. (**f**) Schematic device structures of Au/BM-SCO/LSMO/LAO(001) and Au/BM-SCO/LSMO/STO(001) devices, I–V cycle characteristics, operational voltage, and on/off ratio accumulation plots under 50 voltage scan cycles. (**g**) Migration barrier for oxygen ions within in-plane octahedral and across-tetrahedral paths for the two devices. (**h**) Schematic illustration of the conduction mechanism in the Au/BM-SCO/LSMO/STO(001) device. (**i**) Schematic illustration of the conduction mechanism in the Au/BM-SCO/LSMO/LAO(001) device [83]. Copyright 2022, American Chemical Society. (**j**) Variation of average on/off ratio in Au/radiation BM-SCO/LSMO/STO(001) devices in the as-fabricated state and under different doses of He irradiation. (**k**) Schematic illustration of in-plane uniaxial zero strain, moderate tensile strain, and excessive tensile strain induced by He ion irradiation, where red and gray spheres represent O and Co atoms, respectively, and the black dashed square represents native oxygen vacancies in the SCO film [84]. Copyright 2022, AIP Publishing.

Petrie et al. investigated the influence of tensile strain on the oxygen activation energy by depositing PV-SCO and BM-SCO films on different substrates [82]. As the in-plane tensile stress of the substrate increased, compressive stress appeared out-of-plane, leading to a reduction in the out-of-plane lattice constant of the films. This was reflected in the XRD pattern of PV-SCO, where the (002) peak shifted to higher angles, as shown in Figure 8a. Through techniques such as XAS, it was discovered that with increasing tensile stress, the value of x in SrCoO_x_ decreased (Figure 8c), indicating an increase in oxygen vacancy content. In terms of unit cell volume (Figure 8b), an increase in in-plane tensile stress resulted in an enlargement of the unit cell volume, with a greater change observed in films with a higher oxygen vacancy content.

Using DFT simulations, they investigated two key parameters affecting oxygen migration: the first being the enthalpy of oxygen insertion, H_i_, which is the energy required to insert an oxygen atom into a vacancy; the other being the activation energy barrier, E_a_, which represents the energy required for an oxygen atom to migrate along the oxygen vacancy channel from one position to another. The process of oxygen migration is illustrated in Figure 8d, where O^2−^ ions pass through a high-energy point (saddle point, H_saddle_), which is the position of highest energy in the system. According to the equation E_a_ = H_saddle_ − H_i_, it was found that the saddle point, H_saddle_, slightly decreased with the increase in tensile strain, while H_i_ significantly increased with the increase in tensile strain. As a result, the activation energy E_a_ decreased significantly with the increase in tensile strain, as shown in Figure 8e. Specifically, when oxygen ions moved along the [010] direction of the oxygen vacancy channel from one vacancy site to another, E_a_ increased as the strain changed from +2% to −2%. This provides a theoretical basis for using epitaxial strain to regulate the performance of resistive switching devices based on topological phase transition materials.

Xiang et al. utilized PLD to deposit BM-SCO films on substrates with the La_0.7_Sr_0.3_MoO_3_ bottom electrode, such as STO (strain-free) and LAO (compressive strain of −2.74%), creating Au/BM-SCO/LSMO/STO(001) and Au/BM-SCO/LSMO/LAO(001) devices [83]. Their resistive switching performance was tested, as shown in Figure 8f. The device with LAO as the substrate had a smaller on/off ratio compared with the one with STO as the substrate. However, under 50 resistive switching cycles, the LAO device exhibited a more uniform distribution of operational voltage and on/off ratio, demonstrating greater stability. In combination with DFT calculations, migration barriers were calculated for oxygen ion migration along in-plane octahedral layers and across-tetrahedral layers under strained and unstrained conditions, as depicted in Figure 8g. It was found that the migration barrier was relatively lower along the [100] direction (in-plane migration) with values of 0.37 eV for Au/BM-SCO/LSMO/STO and 0.80 eV for Au/BM-SCO/LSMO/LAO. Under compressive strain, both in-plane and out-of-plane migration barriers increased. Figure 8h,i illustrate the resistive switching mechanisms for the two devices. In the LAO device, compressive stress increased the oxygen vacancy formation energy and oxygen ion migration energy, resulting in finer and fewer CFs in LRS compared with the unstrained device. However, in HRS, both devices exhibited a similar behavior. The LAO device had a lower on/off ratio due to the difficulty of oxygen ion migration. Hence, the compressive strain device demonstrated greater stability in performance.

Furthermore, apart from using substrates to induce in-plane strain in epitaxial films, stress can also be induced through irradiation. Xiang et al. employed low-energy helium (He) ion irradiation (5 kV) with varying doses to the SCO film, leading to out-of-plane tensile strain due to the insertion of helium interstitial atoms. The out-of-plane tensile strain increased from 0.447% to 2.785% with the increase in ion dose [84]. The device performance results are shown in Figure 8j, where the on/off ratio increased initially and then decreased with the increase in external 5 kV helium ion dose (initial on/off ratio of 2.94, on/off ratio of 169 for 1 × 10^14^ He/cm^2^, 20.56 for 5 × 10^14^ He/cm^2^, and 14.22 for 1 × 10^15^ He/cm^2^). The mechanism underlying the strain-induced uniaxial strain’s effect on the SCO device resistive switching behavior is depicted in Figure 8k. Under moderate tensile strain (e.g., in the case of 1 × 10^14^ He/cm^2^), the strength of Co-O bonds (bond l_2_) decreases due to the strain, leading to reduced oxygen migration barriers and facilitating oxygen ion migration. However, excessive tensile strain might overstretch the Co-O bonds (bond l_3_), causing neighboring bonds (bond l_4_) to become stronger, hindering oxygen ion migration and leading to a decrease in the device’s on/off ratio. Notably, excessive in-plane strain can result in the formation of nanoscale defects in the direction perpendicular to the plane, while excessive compressive in-plane strain can lead to horizontal defects parallel to the plane. These defects can severely impact electron transport and oxygen diffusion along the film growth direction, thereby affecting the device’s resistive switching performance [85]. Hence, the precise control of epitaxial strain to enhance the performance of topological phase transition memristors has garnered increasing attention among researchers.

In fact, the film thickness of electrodes and switching matrix play a very important role in the switching mechanism. The research on the effect of film thickness on the performance of memristors is more focused on binary and ternary transition metal oxides [86,87]. For example, when studying Ti/HfO_x_ devices, Rahaman et al. [88] used the high oxygen absorption ability of the Ti layer to change the content of oxygen vacancies in the HfO_x_ layer by controlling the thickness of the Ti layer, so as to regulate the performance parameters well, such as V_forming_ and the working current of the device. Similarly, Tong et al. [89] reported the effect of different film thicknesses on the performance of NiFe_2_O_4_ (NFO) memristors. They prepared several NFO films with different thicknesses by controlling the time of film deposition. It was found that the thicker the film, the longer the path to form CFs, the more dispersed the oxygen distribution, the larger the initial V_forming_ of the device, and the worse the cycle stability. If the NFO film is too thin, the defect-assisted tunneling current in the film is easily conducted through the insulating layer, and the cycle stability of the device is also poor. However, there are few studies on the influence of the thickness of the functional layer film on the performance of the topological phase transition memristor, which requires researchers to conduct more extensive and in-depth research.

## 4. Applications of Topological Phase Transition Memristors in Neural Network Computing

Compared with computers based on the traditional von Neumann architecture, the information storage and logical operations in the human brain are integrated, making the brain more efficient at processing vast amounts of data such as high-precision image recognition and machine learning. The efficiency stems from the brain’s ability to simultaneously process and memorize information by reconfiguring synaptic connection strengths (or synaptic weights). Therefore, to simulate the information processing capabilities of the human brain, emulating neural synapses is paramount, involving the simulation of synaptic plasticity. In biological synapses, the connection strength (or synaptic weight) between presynaptic and postsynaptic neurons is modulated by synaptic spikes. For memristors, the conductance or resistance corresponds to synaptic weight, and it can be adjusted by applying electrical stimuli to the electrodes. As a result, memristors have become one of the ideal devices for emulating synaptic functionality.

**Figure 9:**
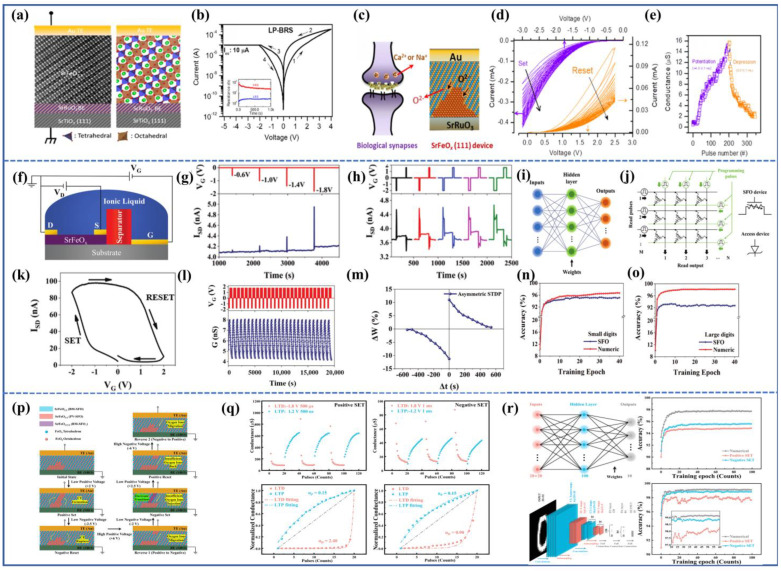
(**a**) Schematic diagram of the device structure Au/BM-SFO/SRO/STO(111). (**b**) I–V characteristic curve under a negative voltage region with ICC limited to 10 μA. (**c**) Schematic representation of the simulated neural synapse of the device Au/BM-SFO/SRO/STO(111). (**d**) Variation in device conductivity under forward voltage scanning and reverse voltage scanning. (**e**) Variation in device conductivity under a series of continuous positive and negative pulse stimulations [90]. Copyright 2020, American Chemical Society. (**f**) Schematic diagram of the three-terminal SFO device based on IL gating. (**g**) Trend of changes in post-synaptic current under different amplitude pre-synaptic pulse stimulation and the read voltage is 0.6 V. (**h**) Trend of changes in post-synaptic current under pre-synaptic pulse stimulation with different widths. (**i**) Typical structure diagram of a multi-layer perceptron (MLP) neural network. (**j**) Hardware schematic diagram of cross-arranged synaptic layers. (**k**) Characteristics curve of the device current versus gate voltage. (**l**) Vg-controlled behavior of long-term potentiation (LTP) and long-term depression (LTD), with Vg pulses consisting of 16 negative pulses (amplitude −1.8V, width 5 s, interval 10 s) and 24 positive pulses (amplitude 1.8 V, width 10 s, and interval 10 s). (**m**) Spike-timing-dependent plasticity (STDP) under the joint action of pre-synaptic and post-synaptic neuron spikes. (**n**) Evolution of training accuracy of the MLP network for 8 × 8 pixel handwritten recognition with training time. (**o**) Evolution of training accuracy of the MLP network for 28 × 28 pixel handwritten recognition with training time [91]. Copyright 2019, Wiley-VCH Verlag GmbH & Co. (**p**) Structure of polar-switchable device Au/BM-SFO/SRO/STO(111) and schematic of polarity switching operation. (**q**) Long-term potentiation (LTP) and long-term depression (LTD) of positive and negative set devices, along with the non-linear factor α. (**r**) Structure of MLP and convolutional neural network (CNN), and evolution of recognition accuracy of positive and negative set devices in both neural network types for the MNIST handwritten dataset, with respect to training time [92]. Copyright 2023, Elsevier B.V.

In the realm of memristor-based synaptic devices, Nallagatla et al. reported a bipolar resistive switching device based on interface barrier-type BM-SFO and observed both long-term potentiation (LTP) and long-term depression (LTD) characteristics [90]. The device structure was Au/BM-SFO/SRO/STO(111), as illustrated in Figure 9a, utilizing Au as the top electrode and SrRuO_3_ as the bottom electrode. The oxygen vacancy planes were oriented outward and connected to the top and bottom electrodes. The I–V characteristics are shown in Figure 9b, exhibiting an asymmetric and gradual resistance transition behavior, likely attributed to the differing Schottky barrier heights at the Au/BM-SFO and BM-SFO/SRO interfaces. This gradual resistance transition feature is more advantageous for applications in emulating neural synapses (as shown in Figure 9c). Following positive and negative voltage sweeps, continuous negative voltage sweeps caused the device’s conductance to gradually increase (corresponding to continuous Set process), while continuous positive voltage sweeps caused the conductance to decrease gradually (corresponding to continuous reset process). Furthermore, when a continuous negative pulse (−4.0 V, 1 ms) was applied to the device, followed by a series of identical positive pulses (5.0 V, 1 ms), and the instantaneous conductance was read out after each pulse using a 0.5 V voltage, the device’s conductance increased gradually under the effect of negative pulses and decreased gradually under the effect of positive pulses, corresponding to LTP and LTD functionality, as shown in Figure 9d,e, which indicates that the device can be well-suited for applications in emulating neural synapses.

Ge et al. utilized ion liquid gating to fabricate a unique three-terminal SFO synaptic device, simulating synaptic plasticity and spike-timing-dependent plasticity (STDP) [91]. The device structure is shown in Figure 9f, and the variation in device conductivity is controlled by the gate voltage. When a negative voltage is applied to the gate, oxygen ions from the ionic liquid enter SFO, which causes the transition from BM-SFO to PV-SFO and, thus, increases device conductivity. Conversely, the transition from PV-SFO to BM-SFO decreases device conductivity, as shown in Figure 9k. The I–V transfer curve exhibits clear hysteresis, indicating excellent reversibility of the device conductivity.

The key to transmitting information in neurons is the excitatory postsynaptic current (EPSC) triggering. To evaluate synaptic response in the device, a series of pre-synaptic pulse V_G_ was applied to observe changes in the post-synaptic current I_SD_. By increasing the amplitude and width of pre-synaptic pulses, the post-synaptic current increased. The device’s LTP and LTD characteristics were subsequently measured. Applying a series of negative pulses with amplitudes of −1.8 V and widths of 5 s and positive pulses with amplitudes of +1.8 V and widths of 10 s to the gate revealed continuous increases and decreases in device conductivity, reflecting reproducible and non-volatile synaptic plasticity. Additionally, STDP is a fundamental Hebbian learning rule used to simulate synaptic functionality. It involves changes in synaptic weights resulting from the interaction of pre-synaptic and post-synaptic neuron spikes. The device’s weight change is shown in Figure 9m. If the pre-synaptic neuron spike precedes the post-synaptic neuron spike (i.e., Δt > 0), LTP occurs, increasing the synaptic weight. Conversely, if the pre-synaptic neuron spike follows the post-synaptic neuron spike (Δt < 0), LTD occurs, decreasing the synaptic weight.

For exploring the performance of artificial neural networks, a typical multi-layer perceptron (MLP) neural network was constructed, as shown in Figure 9i. Training was conducted using two datasets: the Optical Recognition of Handwritten Digits (ORHD) dataset (8 × 8 pixels) and the MNIST dataset (28 × 28 pixels). It was found that for the small dataset, the recognition accuracy of the neural network increased to 95.2% with training, close to the theoretical value of 96.7%. For the larger dataset, the recognition accuracy reached 92.7%.

Furthermore, Su et al. reported a polarity-switchable device based on BM-SFO(111) and studied the device’s LTP, LTD, and its application in the MNIST handwritten recognition scenario [92]. The term “polarity-switchable” refers to the ability to switch the device’s operation polarity. After the initial forming process of the Au/BM-SFO/SRO/STO(111) device, a portion of oxygen ions from the air is attracted to the SFO thin film, forming an oxygen-rich ion cluster near the bottom electrode, as shown in Figure 9p. The device is then set positively and reset negatively. Applying a large positive polarity-switching voltage attracts the oxygen-rich ion cluster to the top electrode, setting the device negatively and resetting it positively. The polarity switching is reversible. The LTP and LTD of the device before and after polarity switching were studied, and the read voltage is 10 mV. The LTD of the negative set device was found to have a smaller non-linear factor α_D_ than the positive set device, indicating that the negative set device better models synaptic plasticity. MLP and convolutional neural network (CNN) structures were subsequently built and tested for handwritten recognition. The negative set device achieved higher recognition accuracy than the positive set device in both network types.

In general, CF-based memristors have characteristics like a large on/off ratio, non-volatility, and low power consumption. Interface switching devices possess traits such as multi-resistance states, no forming process, and low operating voltage. Chen et al. combined these two device characteristics to construct a spiking neural network (SNN) [93]. The CF-based memristor can exploit the transient connection and disconnection of its CF. Time-separated pre- and post-pulses were used to replace the joint action of pre-synaptic and post-synaptic neuron spikes, resulting in device STDP. The interface switching device’s multi-valued characteristics were used to extract a Leaky Integrate-and-Fire (LIF) model. The SNN circuit composed of CF-based memristors as synapses and interface switching devices as neuron modules was constructed. The circuit had read and write modes. When the enable signal from PG_E_ had no pulse, the circuit was in the write module, and the synaptic signal (conductance) from memristor1 was weighted and applied to memristor2 to increase G_M2_. When the enable pulse from PG_E_ appeared, the circuit was in the read state, no longer influenced by synaptic writing, and if G_M2_ was sufficiently large, V_o_ would output a spike pulse to other neurons. The spike pulse would also be applied back to the synapse formed by memristor1 as a post-synaptic neuron spike, modifying the synaptic weight (conductance) according to STDP rules. Finally, SNN was applied to the ORHD handwritten recognition scenario, achieving an accuracy of 83.7% when the number of output neurons was 400. This demonstrates that SFO as a multi-functional material can be well applied to neuron and synaptic devices, aiding in the development of fully memristive SNN.

**Figure 10:**
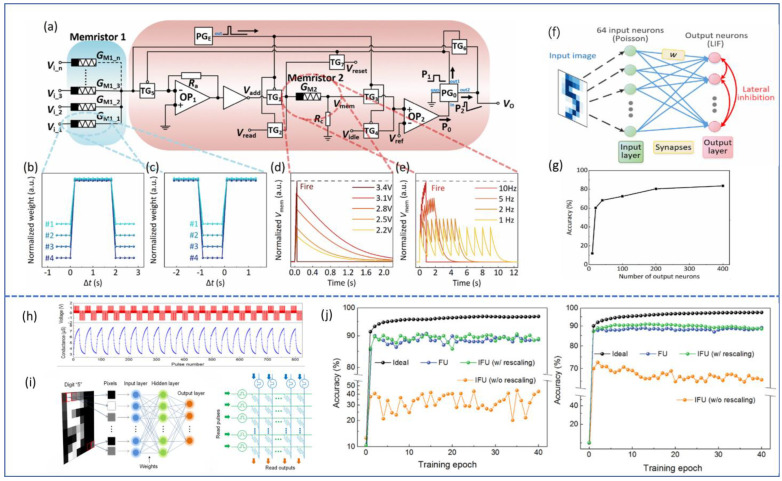
(**a**) Circuit schematic composed of CF-based memristor1 as a synapse and interface switching memristor2 as a neuron. (**b**,**c**) STDP characteristics of memristor1. (**d**,**e**) LIF characteristics of memristor2, and the read voltage is 0.2 V. (**f**) SNN neural network structure and its application in recognizing the ORHD handwritten dataset. (**g**) Relationship between handwritten recognition accuracy and the number of output neurons [93]. Copyright 2022, Elsevier B.V. (**h**) LTP and LTD characteristics of interface switching devices under continuous positive and negative pulse stimulation, and the read voltage is 0.5 V. (**i**) MLP network structure composed of interface switching devices and its application in recognizing the ORHD and MNIST handwritten datasets. (**j**) Relationship between small image and large image recognition accuracy and training cycles for FU, IFU (unscaled), and IFU (rescaled) [63]. Copyright 2021, Elsevier Ltd.

Lastly, Rao et al. reported the application of interface switching devices based on the configuration shown in Figure 4c in neural networks, observing LTP/LTD, EPSC, paired-pulse facilitation (PPF), STDP, and applications in handwriting recognition domain [63]. As depicted in Figure 10h, under continuous positive pulses, the device exhibits LTP, while under continuous negative pulses, it exhibits LTD. Furthermore, when subjected to a single positive pulse, the device current reaches its peak and then decays, demonstrating the process of EPSC. The final stable current remains higher than the initial current, suggesting that part of the weight (conductance) change is volatile, while the rest is non-volatile. Additionally, when two consecutive pulses are applied, the second EPSC is generally higher than the first, and as the time interval Δt between the two pulses decreases, the paired-pulse facilitation (PPF) index increases. Both behaviors can be well fitted by a double-exponential function, demonstrating good PPF. Similarly, by modulating the time interval between presynaptic and postsynaptic pulses, the STDP characteristics of the device are obtained. Subsequently, the MLP network composed of this device is applied to the ORHD and MNIST handwritten recognition datasets, as shown in Figure 10i,j. It is found that after training with small images (ORHD) for 40 cycles, an accuracy of 88.7% is achieved in the frequent weight updates (FU) scenario, which is only about 8.0% lower than the ideal accuracy. However, in the infrequent weight updates (IFU) scenario without rescaling, the accuracy drops to 31.9%. After rescaling, the accuracy in the IFU scenario reaches 89.1%. In the case of large images training, the accuracies achieved in the FU, unscaled IFU, and rescaled IFU scenarios are 88.4%, 65.9%, and 88.8%, respectively. This suggests that interface-switching memristors based on SFO hold significant potential for neural-morphic computing applications [63].

## 5. Conclusions and Outlook

Table 1 presents a comparative analysis of typical memristors based on topological phase transition materials and other representative memristors in terms of the switching ratio, retention characteristics, cycling properties, LTP/LTD, STDP, PPF, and handwriting recognition. Among them, memristors based on typical topological phase transition materials like SrFeO_x_ and SrCoO_x_ do not change their lattice framework during resistive switching, making the resistive switching process clearer, and the retention characteristics and cycle to cycle characteristics of the devices are better. The controllable variation of oxygen stoichiometry enables diverse resistive switching behaviors in memristor devices, such as CF-type devices and interface switching-type devices, better simulating the functions of neural synapses and neurons.

For resistive memories, electrode engineering, strain engineering, ionic liquid gating, and other approaches are effective means to modulate the resistive switching performance of devices. Despite substantial research progress in recent years, the stochastic nature of CF generation and rupture during the resistive switching process still adversely affects both the internal cycling of devices and their uniformity across devices. The fabrication process of topological phase transition memristors is complex, sensitive to oxygen content, and incompatible with the traditional CMOS process, which also restricts its development. Therefore, the study of interface-type memristors, the introduction of dominant growth sites of conductive filaments by doping or regulating the interface defects of top and bottom electrodes, and the combination with more mature binary metal oxide materials need to be further developed to meet the needs of high switching ratio, high uniformity, and high endurance of memristors.

Currently, memristors based on topological phase transition materials, such as CF-type devices, offer characteristics like high switching ratios and rapid state switching, making them suitable for simulating neural synapses in SNNs. Meanwhile, interface switching devices, due to their excellent multi-state characteristics, can simulate neurons in SNNs. This suggests the potential to construct an all-memristor SNN network. Furthermore, memristor arrays exhibit high applicability in CNNs, MLP networks, and other areas, promising extensive use in image processing, artificial intelligence, autonomous driving, and more. However, the main limiting factor in the development of neuromorphic devices is the relatively low linearity of memristors. In the future, simulating the ion dynamics within synapses using interface-switching memristors or constructing new memristor resistances or arrays may further advance the development of artificial neural-morphic networks.

It is worth noting that materials like SrFeO_x_ and SrCoO_x_ undergo a transition from insulating perovskite to conductive perovskite phases, corresponding to a transition from antiferromagnetic to ferromagnetic states. This indicates the potential application of topological phase transition materials in spintronic devices. Lastly, materials like PV-SCO with low overpotentials, which can be stabilized in a cubic crystal structure by incorporating scandium (Sc) and niobium (Nb) into their B-site, show significantly enhanced electrocatalytic oxygen evolution activity [94]. Thus, topological phase transition perovskite materials possess broad potential applications in fields such as electronics, magnetism, materials science, and informatics, serving interdisciplinary technological needs.

In conclusion, we have provided a detailed overview of topological phase transition perovskite memristors, covering their structure, device fabrication, performance modulation, simulation of neural synapses and neurons, and applications in neuromorphic computing. The strong coupling between charge, spin-orbit, and lattice structures in topological phase transition perovskite materials endows them with favorable electrical, magnetic, and optical properties, promising applications in memristors, integrated optical devices, gas sensors, and beyond. Nonetheless, the commercial application of memristors based on topological phase transition materials still faces challenges, and requires continuous dedication and focused research efforts from scientists and researchers.

## Figures and Tables

**Table 1:** Comparison of resistive switching performance between memristors based on topological phase-change materials and other representative memristors.

Device Structure	On/Off Ratio	Retention [s]	Endurance [Cycle]	LTP/ LTD	STDP	PPF	Conductive Type	Neural Network Performance
Au/SrFeO_2.5_/SrFeO_3_/SrRuO_3_ [63]	25	-	-	√	√	√	I-RRAM	83.7% (8 × 8)
Au/SrCoO_2.5_/SrRuO_3_ [95]	~10^2^	3000 s	10^8^	√	-	-	CF-RRAM	-
Au/SrFeO_2.5_/SrRuO_3_ [90]	~10^2^	1000 s	-	√	-	-	CF-RRAM	90.5% (28 × 28)
Au/SrFeO_2.5_/SrRuO_3_ [92]	40	5000 s	-	√	-	-	CF-RRAM	98.81% (28 × 28)
Au/BM-SFO/Nb:STO [96]	10^4^	2000 s	10^3^	-	-	-	CF-RRAM	-
Au/Nb:STO/Au [60]	10^5^	10^4^ s	10^2^	√	√	√	I-RRAM	94.72% (28 × 28)
Pt/BiFeO_3_/HfO_2_/TiN [97]	10^4^	10^4^ s	10^8^	√	√	-	CF-RRAM	-
Pt/Li_4_Ti_5_O_12_/TiO_2_/Pt [98]	10	10^4^ s	-	√	-	-	CF-RRAM	87% (20 × 20)
TiN/TaO_2_/Pt [99]	5	10^4^ s	10^3^	√	-	-	I-RRAM	93.25% (28 × 28)
Mo/TiOx/TiN [100]	~10^2^	-	-	-	-	-	I-RRAM	90% (28 × 28)
Pt/TaOx/HfO_2_/TiN [101]	10^2^	10^4^ s	300	-	-	-	I-RRAM	80.8% (28 × 28)
Ni/Ta_2_O_5_/Si [102]	10^3^	10^4^ s	10^3^	√	√	√	I-RRAM	86.3% (28 × 28)
Ag/SiNx/a-Si [103]	20	10^3^ s	500	√	-	-	I-RRAM	91.3% (28 × 28)

## Data Availability

Not applicable.
